# Chitinase-3-like 1 is a biomarker of acute kidney injury and mortality in paediatric severe malaria

**DOI:** 10.1186/s12936-018-2225-5

**Published:** 2018-02-15

**Authors:** Andrea L. Conroy, Michael T. Hawkes, Robyn Elphinstone, Robert O. Opoka, Sophie Namasopo, Christopher Miller, Chandy C. John, Kevin C. Kain

**Affiliations:** 1 Department of Pediatrics, Indiana University School of Medicine, 1044 West Walnut St., Building 4, Indianapolis, IN 46202 USA; 2 Sandra Rotman Centre for Global Health, Toronto General Hospital, University Health Network, MaRS Centre, 101 College St. TMDT 10-360A, Toronto, ON M5G 1L7 Canada; 3 Division of Infectious Diseases, Department of Medicine, University of Toronto, Toronto, ON Canada; 4 Division of Pediatric Infectious Diseases, 3-593 Edmonton Clinic Health Academy, University of Alberta, Edmonton, AB T6G1C9 Canada; 5 Department of Pediatrics and Child Health, Makerere University, Kampala, Uganda; 6 Department of Pediatrics, Jinja Regional Referral Hospital, P.O. Box 43, Jinja, Uganda; 7 University of British Columbia, Vancouver, Canada

**Keywords:** Paediatric, Severe malaria, Acute kidney injury, Chitinase-3 like 1, Nitric oxide therapy, Adjunctive therapy, Mortality, Inflammation, Endothelium, Hemolysis

## Abstract

**Background:**

Chitinase-3-like 1 (CHI3L1) is a glycoprotein elevated in paediatric severe malaria, and an emerging urinary biomarker of acute kidney injury (AKI). Based on the hypothesis that elevated CHI3L1 levels in malaria are associated with disease severity, the relationship between plasma CHI3L1 levels, AKI and mortality was investigated in Ugandan children enrolled in a clinical trial evaluating inhaled nitric oxide (iNO) as an adjunctive therapy for severe malaria.

**Methods:**

Plasma CHI3L1 levels were measured daily for 4 days in children admitted to hospital with severe malaria and at day 14 follow up. AKI was defined using the Kidney Disease: Improving Global Outcomes consensus criteria. This is a secondary analysis of a randomized double-blind placebo-controlled trial of iNO versus placebo as an adjunctive therapy for severe malaria. Inclusion criteria were: age 1–10 years, and selected criteria for severe malaria. Exclusion criteria included suspected bacterial meningitis, known chronic illness including renal disease, haemoglobinopathy, or severe malnutrition. iNO was administered by non-rebreather mask for up to 72 h at 80 ppm.

**Results:**

CHI3L1 was elevated in patients with AKI and remained higher over hospitalization (p < 0.0001). Admission CHI3L1 levels were elevated in children who died. By multivariable analysis logCHI3L1 levels were associated with increased risk of in-hospital death (relative risk, 95% CI 4.10, 1.32–12.75, p = 0.015) and all-cause 6 month mortality (3.21, 1.47–6.98, p = 0.003) following correction for iNO and AKI. Treatment with iNO was associated with delayed CHI3L1 recovery with a daily decline of 34% in the placebo group versus 29% in the iNO group (p = 0.012). CHI3L1 levels correlated with markers of inflammation (CRP, sTREM-1, CXCL10), endothelial activation (Ang-2, sICAM-1) and intravascular haemolysis (LDH, haem, haemopexin).

**Conclusions:**

CHI3L1 is a novel biomarker of malaria-associated AKI and an independent risk factor for mortality that is associated with well-established pathways of severe malaria pathogenesis including inflammation, endothelial activation, and haemolysis.

*Trial registration* Clinicaltrials.gov, NCT01255215. Registered December 7th 2010

**Electronic supplementary material:**

The online version of this article (10.1186/s12936-018-2225-5) contains supplementary material, which is available to authorized users.

## Background

Acute kidney injury (AKI) is an important complication in severe malaria that is associated with increased mortality in both paediatric [1–6] and adult populations [7–10]. However, until recently, the incidence of AKI in paediatric malaria was under-appreciated as few children develop signs of overt renal failure and creatinine testing is not routinely available in resource-constrained settings. Despite significant progress in reducing malaria incidence and mortality [11], case fatality rates in severe disease remain high, and the identification of an effective adjunctive therapy is a research priority.

Decreased bioavailable nitric oxide is a common feature in both children and adults with severe malaria, and clinical trials to increase nitric oxide (NO) have been conducted [12–14]. Although there has been no conclusive benefit demonstrated in human trials designed to increase bioavailable NO, infusion of l-arginine in adults with severe malaria improved endothelial recovery [12], and iNO delivered at 80 ppm was associated with reduced risk of fine motor impairment in children under 5 years of age at 6 month follow up [15].

Despite early reports that inhaled NO (iNO) was associated with increased splanchnic and renal blood flow [16], a meta-analysis of adults with acute respiratory distress syndrome found that iNO treatment was associated with a 50% increased risk of developing AKI (relative risk, 95% CI 1.50, 1.11–2.02) [17]. This has been confirmed in another meta-analysis including non-ARDS patients [18], but the effect was strongest in patients with ARDS with prolonged exposure and a high cumulative dose. There are limited reports evaluating iNO and renal safety in children. Previously, an increase in the overall incidence of AKI (relative risk, 95% CI 1.36, 1.03–1.90, p = 0.026) was reported in children treated with iNO [6]. However, there was no association between iNO and AKI when restricting the analysis to children who developed AKI after treatment was initiated.

Chitinase-3-like 1 protein (CHI3L1) is a 39 kDa secreted glycoprotein produced by a variety of cell types in response to inflammation, including activated macrophages, neutrophils, and fibroblasts. CHI3L1 is highly expressed in healthy kidney tissue [19] and is freely filtered by the glomerulus. CHI3L1 is also secreted by activated macrophages in the kidney upon stress or damage [20]. Elevated levels of CHI3L1 have been reported in Ugandan children with severe malaria, and further elevated in fatal malaria [21]. Co-culture of human peripheral blood mononuclear cells with *Plasmodium falciparum*-infected erythrocytes in vitro induced CHI3L1 transcription and secretion of CHI3L1 protein [21]. While CHI3L1 was elevated by day 5 infection in an experimental model of cerebral malaria, genetic disruption of *Chi3l1* did not affect inflammatory responses or outcome [21].

In this secondary analysis of an iNO intervention trial, CHI3L1 was investigated as a biomarker of morbidity and mortality in paediatric severe malaria, the longitudinal kinetics of CHI3L1 were explored in children hospitalized with severe malaria, and the impact of iNO therapy on CHI3L1 normalization was evaluated.

## Methods

### Study design

The study was conducted between 2011 and 2013 at the Jinja Regional Referral Hospital in Jinja, Uganda. All children were treated with intravenous artesunate followed by oral artemisinin-based combination therapy. Inclusion criteria were age 1–10 years, *P. falciparum* by RDT (First Response Malaria Ag. HRP2/pLDH Combo Rapid Diagnostic Test, Premier Medical Corporation Limited, India), selected severe malaria criteria (decreased consciousness, repeated seizures, prostration, and/or respiratory distress), and plasma sample available for CHI3L1 testing. Exclusion criteria included: known chronic illness, severe malnutrition, known haemoglobinopathy, prior treatment with quinine in the emergency department, suspicion of acute bacterial meningitis. Study gas was delivered continuously by non-rebreather mask for up to 72 h, as previously described [13, 22].

### Acute kidney injury

Creatinine and BUN were assessed at the point of care using i-STAT CHEM8+ or Crea cartridges (Abbott Laboratories, Saint-Laurent, Québec). Creatinine measured by i-STAT is calibrated traceable to the isotype dilution mass spectrometry reference measurement and is free of interference from haemoglobin, bilirubin, and glucose [23]. Estimated glomerular filtration rate (eGFR) was calculated using the Bedside Schwartz equation, using a constant for children (k = 0.413) [24]. Presence of AKI was determined retrospectively using KDIGO guidelines [25]. Children were considered to have AKI if they had either a ˃26 μmol/L rise in creatinine within 48 h or > 1.5-fold increase in creatinine from estimated baseline. Baseline creatinine was estimated assuming a normal GFR of 120 mL/min/1.73 m^2^ and using the Schwartz equation to back calculate creatinine based on the child’s height. The children with AKI were further classified by stage: stage 1 (risk; 1.5–1.9-fold increase in creatinine from nadir), stage 2 (injury; 2.0–2.9-fold increase), and stage 3 (failure; greater than 3.0-fold increase, single value greater than 354 µmol/L during hospitalization, or an eGFR of < 35 mL/min/1.73 m^2^) [25]. The incidence of AKI in this cohort has been previously reported [6].

### Laboratory testing

K_2_ EDTA anticoagulated plasma samples were collected daily from children during hospitalization (day 1–4), and at follow up (day 14) and stored at − 80 °C until testing. Cystatin C and CHI3L1 were measured by ELISA (DuoSet, R&D Systems, Burlington, Canada) with the investigator blinded to treatment group and outcome [6]. LDH activity was measured using a colorimetric assay according to manufacturer’s protocol (BioVision, Milpitas, CA, USA). The limit of detection for CHI3L1 was 4.5 ng/mL with inter- and intra-assay reproducibility of 5.9 and 6.3%, respectively. Markers of intravascular haemolysis (haem, haemopexin) [26], endothelial activation (Ang-2, sICAM-1) [27], and inflammation (CRP, sTREM-1, CXCL10/IP-10) were measured by ELISA as previously described [27, 28]. Biomarkers were selected for analysis based on an established association with disease severity and mortality in Ugandan children with severe malaria [27, 28].

### Statistical analysis

Data were analysed using Stata/SE v14.0, GraphPadPrism v7.03, and R [29]. For access to the dataset see Additional file 1. Continuous data are presented as median (interquartile range, IQR) and analyzed using Wilcoxon rank sum test or non-parametric test for trend. Categorical data are presented as n (%) and analysed using Pearson’s Chi Square or Fisher’s exact test, as appropriate. To compare biomarker levels at admission, Spearman’s non-parametric correlation was used. To assess the relationship between CHI3L1 and mortality, generalized linear models were used opting first for a log-binomial model with robust standard errors. In the event of failed convergence Poisson regression was used with robust standard errors.

R [29] and lme4 [30] were used to perform a linear mixed effects (LME) analysis of in-hospital longitudinal course of log (CHI3L1) over time in patients without AKI and with different stages of AKI. Time, nitric oxide treatment arm, and AKI stage were entered as fixed effects with and without interaction terms. Intercepts and slopes were modeled for each subject as random effects. The intercept for treatment arm was constrained to zero at baseline as children were randomly allocated to treatment arm. Visual inspection of residual plots did not reveal deviations from homoscedasticity or normality. p values were obtained by likelihood ratio tests of the full model (including AKI stage) against the model without AKI stage.

## Results

### CHI3L1 levels at presentation are associated with disease severity and AKI

**Fig. 1:**
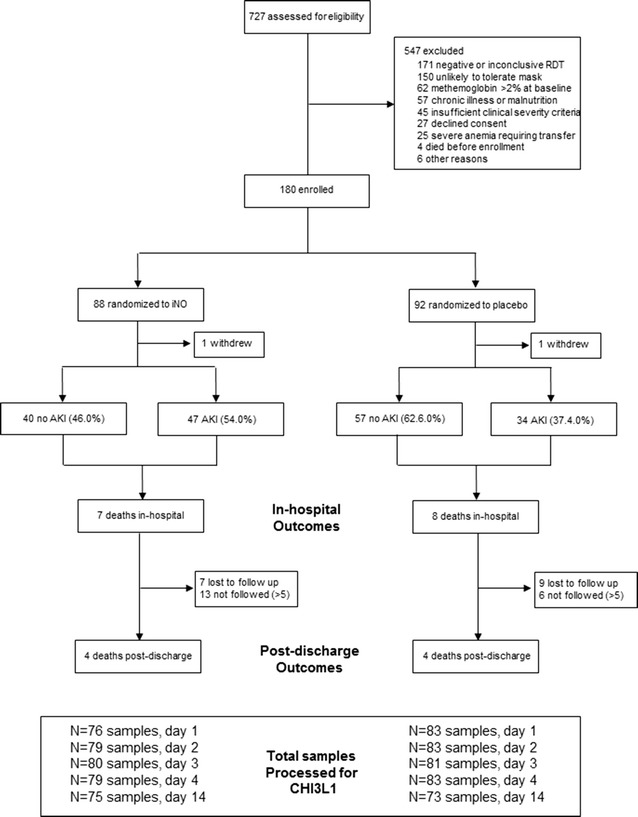
Flow chart of study population

Levels of CHI3L1 were available for 159 children at admission (Fig. 1). A description of the population is included in Table 1. The median age of children was 2.0 years and 56% were male. At admission, children with CHI3L1 levels in the highest quartile had a median of 5 severe malaria criteria compared to 4 in children with the lowest three quartiles (p < 0.0001) [31]. Children with elevated CHI3L1 also had higher lactate (p = 0.001), lower bicarbonate, (p < 0.0001) and higher Cystatin C (p = 0.001) and BUN levels (p = 0.025). Children with CHI3L1 levels in the highest quartile were more likely to have AKI (p = 0.034), and there was a strong relationship between CHI3L1 and severity of AKI with 5.9% of children with CHI3L1 levels in the lowest three quartiles having stage 3 AKI compared to 28.2% of children with CHI3L1 levels in the highest quartile having stage 3 AKI (p < 0.0001). The relationship between CHI3L1 levels and kidney function was further explored using linear regression to investigate the relationship between creatinine, Cystatin C. and BUN as dependent variables and log_10_(CHI3L1) levels at admission as the independent variable (Table 2). A log_10_ increase in CHI3L1 was significantly associated with creatinine, Cystatin C, and BUN following adjustment for age and sex. When adjusting for other measures of kidney function, log_10_(CHI3L1) remained independently associated with an increase in Cystatin C following correction for creatinine (beta, 95% CI 0.17, 0.05–0.30, p = 0.008), but not BUN. The relationship between log_10_(CHI3L1) and BUN was not significant when adjusting for either creatinine or Cystatin C (p > 0.05), and the relationship between log_10_(CHI3L1) and creatinine was not significant when adjusting for Cystatin C or BUN (p > 0.05).

**Table 1:** Patient characteristics based on quartiles of CHI3L1 levels at admission

	Cohort	CHI3L1		Q123 versus Q4, p value
		Q123 (n = 120)	Q4 (n = 39)	
Patient demographics				
Age, years	2.0 (1.0, 3.0)	2.0 (1.0, 3.0)	2.0 (1.0, 3.0)	0.825
Sex, males	89 (56.0)	68 (56.7)	21 (53.8)	0.450
Weight, kg	11.0 (9.0, 13.0)	11.0 (9.0, 13.0)	11.0 (9.0, 13.0)	0.893
Height, cm	79 (71, 90)	79 (71, 90)	80 (70, 88)	0.850
Weight-for-age z	− 1 (− 2, 0)	− 1 (− 2, 0)	0 (− 2, 0)	0.247
Height-for-age z	− 2 (− 3, 0)	− 2 (− 3, 0)	− 2 (− 3, − 1)	0.575
Clinical parameters at admission				
Temperature	37.9 (37.0, 38.8)	38.0 (37.0, 39.0)	37.8 (37.0, 38.4)	0.304
Heart rate	161 (144, 179)	161 (142, 177)	162 (153, 181)	0.186
Respiratory rate	48 (38, 62)	46 (36, 60)	55 (44, 66)	0.056
Systolic BP	110 (100, 120)	110 (100, 120)	110 (100, 125)	0.740
Diastolic BP	60 (50, 70)	60 (50, 70)	60 (50, 70)	0.932
Coma	95 (59.7)	67 (55.8)	28 (71.8)	0.077
Convulsions	126 (79.2)	95 (79.2)	31 (79.5)	0.966
Severe anaemia	104 (65.4)	74 (61.7)	30 (76.4)	0.082
Haemoglobinuria	26 (16.4)	20 (16.7)	6 (15.4)	0.403
Jaundice	25 (15.8)	17 (14.3)	8 (20.5)	0.355
Deep breathing	74 (46.5)	54 (45.0)	20 (51.3)	0.494
Shock	14 (8.8)	9 (7.5)	5 (12.8)	0.308
Severe malaria criteria	4 (3, 6)	4 (3, 5)	5 (4, 6)	0.0004
Laboratory tests				
Parasitaemia	25,280 (2620, 78,840)	23,000 (2640, 72,320)	36,320 (2525, 115,600)	0.264
Lactate, μmol/L	3.6 (2.1, 6.5)	3.1 (2.0, 5.5)	5.5 (3.0, 10.3)	0.001
Glucose, μmol/L	6.7 (5.6, 8.1)	6.8 (5.8, 8.1)	6.3 (4.2, 8.5)	0.106
Haemoglobin, g/dL	4.7 (3.0, 6.4)	4.8 (3.0, 6.7)	4.6 (3.1, 5.4)	0.341
WBC	11.6 (7.5, 19.4)	11.30 (7.65, 19.30)	12.50 (6.70, 20.30)	0.746
Platelets × 10^3^	71 (38, 124)	73 (42, 133)	60 (29, 114)	0.186
HCO3^−^	17.3 (13.0, 20.1)	18.3 (14.8, 20.2)	12.4 (10.5, 18.0)	0.0001
Na^+^	137 (135, 140)	138 (135, 140)	137 (134, 140)	0.289
K^+^	4.1 (3.7, 4.5)	4.1 (3.7, 4.4)	4.3 (3.8, 4.9)	0.125
Cl^−^	108 (104, 112)	108 (104, 112)	110 (105, 114)	0.501
Creatinine, μmol/L	31 (23, 41)	30 (24, 38)	34 (23, 58)	0.260
Cystatin C	1041 (778, 1313)	954 (754, 1191)	1247 (1054, 1545)	0.001
BUN	16.0 (9.0, 27.5)	14.0 (9.0, 25.0)	25.5 (10.8, 42.0)	0.025
Treatment group				
Received iNO	76 (47.8)	53 (44.2)	23 (59.0)	0.108
Acute kidney injury				
AKI	70 (44.3)	47 (39.5)	23 (59.0)	0.034
AKI stage				
0	88 (55.7)	72 (60.5)	16 (41.0)	< 0.0001
1	38 (24.1)	32 (26.9)	6 (15.4)	
2	14 (8.9)	8 (6.7)	6 (15.4)	
3	18 (11.4)	7 (5.9)	11 (28.2)	
Incident AKI^a^	22 (13.9)	17 (14.3)	5 (12.8)	0.819
Outcome				
Discharge with disability	7 (6.7)	6 (7.2)	1 (4.6)	1.000
Death, in-hospital	14 (8.8)	7 (5.8)	7 (18.0)	0.020
Death, 6 months^b^	22 (17.3)	13 (13.5)	9 (29.0)	0.048

Data presented as median (interquartile range) or n (%). Continuous data analyzed using Mann–Whitney U test and dichotomous variables analysed using Pearson’s Chi Square or Fisher’s exact test

^a^Incident AKI defined as AKI that developed following admission to hospital (n = 55 cases of AKI were present on admission)

^b^Outcome available for 127 children

**Table 2:** Relationship between log_10_CHI3L1 levels and kidney function

Dependent variable	Unadjusted beta (95% CI)	p value	Adjusted beta^a^ (95% CI)	p value
Creatinine	13.97 (5.16, 22.77)	0.002	13.51 (4.66, 22.36)	0.003
Cystatin C	0.28 (0.13, 0.44)	< 0.0001	0.28 (0.13, 0.44)	< 0.0001
BUN	10.21 (3.92, 16.51)	0.002	10.23 (3.87, 16.60)	0.002

^a^Adjusted for age and sex

### Admission CHI3L1 is independently associated with in-hospital and all-cause 6 month mortality

CHI3L1 levels were compared at admission with subsequent in-hospital mortality and all-cause 6 month mortality. CHI3L1 levels in the highest quartile were significantly associated with death (Table 1). Further, median CHI3L1 levels were higher among children who died in-hospital than those who survived (p = 0.023, Wilcoxon rank sum test) and children who died by 6 months follow up compared to those known to survive (p = 0.046). By multivariable analysis, a one unit increase in log_10_(CHI3L1) was associated with a 4.10-fold increased risk of in-hospital death (95% CI 1.32–12.75, p = 0.015) following adjustment for treatment arm and AKI status using a log binomial model. Further, a one unit increase in log_10_(CHI3L1) was associated with a 3.21-fold increased risk of death by 6 months (95% CI 1.47–6.98, p = 0.003) following adjustment for treatment arm and AKI status.

Survival data were not available at 6 months follow up for 16 children lost to follow up (LTFU) and 19 children > 5 years of age and not followed to 6 months (according to the study protocol). To evaluate whether the association between CHI3L1 and 6 month mortality was robust to various assumptions a sensitivity analysis was performed first assuming all children < 5 years of age LTFU died (Table 3, model 1). Then all children < 5 years of age LTFU were assumed to have survived (model 2). Assuming all children < 5 years of age LTFU died, log_10_(CHI3L1) was associated with a 2.13-fold increased risk of death (1.18, 3.84, p = 0.012, model 1), and a 3.11-fold increased risk of death (1.38–6.99, p = 0.006, model 2) if all children < 5 years of age LTFU survived. This analysis was then expanded to all children in the cohort (including children > 5 years of age that were not followed). Assuming all children LTFU or > 5 years of age died, log_10_(CHI3L1) was associated with a 1.99-fold increased risk of death by 6 months (1.22–3.25, p = 0.006, model 3). Assuming all children LTFU or > 5 years of age survived, log_10_(CHI3L1) was associated with a 3.10-fold increased risk of death by 6 months (1.34–7.16, p = 0.008, model 4).

**Table 3:** Association between admission CHI3L1 levels and risk of death

	In-hospital mortality		All cause 6 month mortality	
	RR (95% CI)	p value	RR (95% CI)	p value
Primary models				
iNO group	0.72 (0.28, 1.90)	0.512	0.85 (0.41, 1.76)	0.670
AKI	2.61 (0.85, 8.00)	0.094	1.28 (0.61, 2.68)	0.521
Log_10_CHI3L1	4.10 (1.32, 12.75)	0.015	3.21 (1.47, 6.98)	0.003
Sensitivity analysis				
Model 1: children < 5 years of age LTFU died^a^				
iNO group	–	–	0.87 (0.52, 1.45)	0.589
AKI	–	–	1.33 (0.76, 2.32)	0.312
Log_10_CHI3L1	–	–	2.13 (1.18, 3.84)	0.012
Model 2: children < 5 years of age LTFU survived				
iNO group	–	–	0.78 (0.37, 1.65)	0.513
AKI	–	–	1.29 (0.61, 2.75)	0.505
Log_10_CHI3L1	–	–	3.11 (1.38, 6.99)	0.006
Model 3: children < 5 years of age LTFU or > 5 years died^a^				
iNO group	–	–	0.76 (0.49, 1.17)	0.213
AKI	–	–	1.21 (0.77, 1.90)	0.419
Log_10_CHI3L1	–	–	1.99 (1.22, 3.25)	0.006
Model 4: children < 5 years of age LTFU or > 5 years survived				
iNO group	–	–	0.86 (0.40, 1.84)	0.701
AKI	–	–	1.30 (0.60, 2.81)	0.509
Log_10_CHI3L1	–	–	3.10 (1.34, 7.16)	0.008

Default model: generalized linear model with binomial family and log link

*LFTU* lost to follow up

^a^In the event of failed convergence a Poisson model with robust standard errors was used

### CHI3L1 levels are associated with endothelial activation, inflammation and hemolysis

In order to explore potential pathophysiologic mechanisms linking elevated CHI3L1 and mortality in severe paediatric malaria, admission levels of CHI3L1 were compared with other host markers of immune and endothelial activation. CHI3L1 was correlated with pathways implicated in the pathobiology of severe malaria including markers of endothelial activation (angiopoietin-2, 0.43, p < 0.0001; sICAM-1, 0.43, p < 0.0001), markers of inflammation (CRP, 0.36, p < 0.0001; CXCL10/IP-10, 0.26, p = 0.0008; sTREM-1, 0.23, p = 0.005); and markers of haemolysis (LDH, 0.28, p = 0.0004; haemopexin, − 0.23, p = 0.004; haem, 0.30, p = 0.0001).

### Inhaled nitric oxide is associated with delayed recovery of CHI3L1 levels in children with severe malaria

**Fig. 2:**
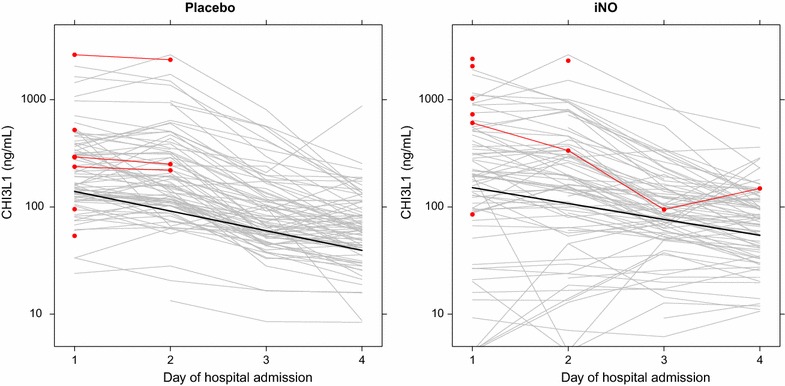
Treatment with inhaled nitric oxide is associated with a slower recovery of CHI3L1 levels. Line plots showing individual CHI3L1 trajectories for patients randomized to receive placebo (left) or inhaled nitric oxide (iNO, right). CHI3L1 levels for in-hospital mortality are depicted in red. The line for the random-intercept, random-slope linear mixed effects model is shown in black. The CHI3L1 concentration (estimate, 95% CI 213 ng/mL, 176–259) decreased by 34% per day (95% CI 31–38) in the placebo group and 29% per day (95% CI 25–33) in the iNO group (p = 0.0071, likelihood ratio test)

LME models were used to explore the relationship between the longitudinal time course of CHI3L1 in patients in the placebo and treatment arms of the iNO trial. Assuming that differences at baseline between trial arms were due to chance alone (random allocation), we observed that CHI3L1 was elevated at baseline and decreased over the first 3 days of hospitalization at a different rate in children receiving iNO compared to placebo. The baseline (day 1) CHI3L1 concentration (estimate, 95% CI 213 ng/mL, 176–259) decreased by 34% per day (95% CI 31–38) in the placebo group and 29% per day (95% CI 25–33) in the iNO group (p = 0.007) (Fig. 2).

### Longitudinal CHI3L1 levels are elevated in patients with AKI, in both placebo- and iNO-treated children with severe malaria

**Fig. 3:**
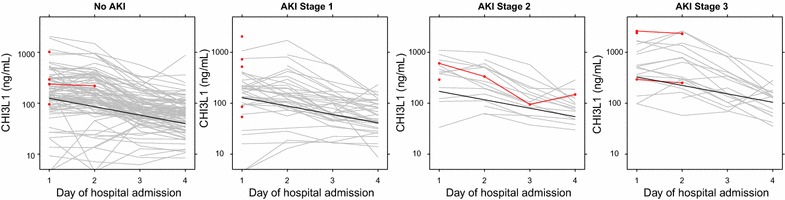
CHI3L1 levels are elevated over hospitalization according to the severity of acute kidney injury. Line plots showing individual CHI3L1 trajectories for patients according to the severity KDIGO-defined acute kidney injury (AKI). CHI3L1 levels for in-hospital mortality are depicted in red. The line for the linear mixed effects model is shown in black. Time, nitric oxide treatment arm, and AKI stage were entered as fixed effects. In this model, AKI grade 1, 2 and 3 was associated with CHI3L1 concentrations 1.02, 1.3, and 2.5-fold higher than patients without AKI, over the course of the first 3 days of hospitalization (p < 0.0001)

AKI is associated with higher mortality in paediatric severe malaria [6]; therefore, the relationship between longitudinal time course of CHI3L1 in patients with and without AKI was investigated. In an analysis including all trial participants and adjusting for the effect of iNO, AKI was associated with higher CHI3L1 levels (1.02-, 1.30-, and 2.50-fold higher in AKI grade 1, 2 and 3, respectively, relative to no AKI, p < 0.0001, Fig. 3). To confirm the association between AKI and elevated CHI3L1 independent of iNO exposure, a subgroup analysis was performed using patients in the placebo arm of the trial: AKI grade 1, 2 and 3 was associated with CHI3L1 concentrations 0.94, 1.31, and 2.41-fold higher than patients without AKI, over the course of the first 3 days of hospitalization (p = 0.017).

## Discussion

Acute kidney injury has recently become recognized as a common complication of paediatric severe malaria, but its pathogenesis is not well understood. In this study, elevated CHI3L1 levels at admission were associated with the severity of AKI. Using linear regression we explore the relationship between CHI3L1 levels and biomarkers of kidney function and found that CHI3L1 was significantly associated with increased Cystatin C following correction for age, sex and creatinine. CHI3L1 levels were associated with an increased risk of both in-hospital and long-term mortality independent of treatment arm and AKI. Importantly, using LME models to evaluate the longitudinal course of CHI3L1, administration of iNO was associated with prolonged elevation of CHI3L1 levels over the first 4 days of hospitalization. Further, there was a strong relationship between CHI3L1 levels and the severity of AKI that was independent of iNO treatment. These results suggest that CHI3L1 is an important biomarker of disease severity and mortality in paediatric severe malaria that is associated with kidney dysfunction as well as endothelial activation, inflammation and haemolysis. Additional studies are required to validate the relationship between CHI3L1 and AKI in severe malaria and investigate the association between CHI3L1 and established biomarkers of AKI.

CHI3L1 is a relatively new biomarker of AKI or altered renal function that has been investigated in the context of critical illness [32, 33], and sepsis [34]. However, CHI3L1 has been predominantly investigated as a biomarker of AKI in urine rather than blood. CHI3L1 was identified in a urine proteomic screen in mice with kidney ischaemic reperfusion injury where a direct correlation was observed between the severity of kidney injury, CHI3L1 expression in the kidney, and levels in the urine [20]. Studies of urinary CHI3L1 from donor kidneys suggested that CHI3L1 is a repair phase protein produced in response to tubular injury, and associated with recovery from AKI and delayed graft function [35]. CHI3L1 expression has been reported on the surface of tubular epithelial cells, consistent with either uptake of filtered CHI3L1 or tubular cell secretion, and urinary CHI3L1 levels correlate with the severity of acute tubular necrosis [35].

Studies in mice have shown that CHI3L1 plays a critical role in tissue repair and remodelling following pulmonary insult by limiting oxidative damage, stimulating alternative (M2) macrophage activation, and inhibiting apoptosis [36–38]. In the context of malaria, an increase of M2 monocytes in peripheral blood has been reported in children with severe malaria compared to healthy controls [39]. Further, M2 monocytes were associated with increased expression of arginase 1, lower NOS2 mRNA, and lower plasma arginine [39]. Additional studies are needed to delineate the role between CHI3L1, alternative macrophage activation in severe malaria and its relationship with AKI and NO bioavailability.

There are limited, and conflicting, data related to the relationship between CHI3L1 and NO. CHI3L1 has been positively correlated with NO levels in tissue culture supernatant from herniated lumbar discs [40], and exhaled NO in children with severe therapy-resistant asthma [41]. However, in patients with type 1 diabetes reduced NO in the blood correlated with elevated CHI3L1 [42], and plasma CHI3L1 was negatively associated with nitric-oxide mediated vasodilatory capacity in adults with obstructive sleep apnea [43]. In this study there was delayed CHI3L1 recovery in children receiving iNO independent of AKI-associated changes in CHI3L1, with CHI3L1 the only biomarker identified that has been shown to differ in response to iNO therapy [27]. While there were differences in the rate of CHI3L1 recovery over the first 4 days of hospitalization associated with iNO therapy, the effect was not significant by day 14 suggesting that iNO results in a transient delay in the normalization of CHI3L1 levels.

Acute kidney injury is a common complication in severe malaria but its pathogenesis is unclear. Peripheral parasitaemia is not associated with worsening kidney function in children or adults with severe malaria [6, 10], but plasma HRP2 levels (reflecting total parasite biomass) are associated with worsening renal function, suggesting AKI is associated with the sequestered parasite biomass [10]. Plasma suPAR—a marker of mononuclear cell activation—was elevated in adults with malaria-associated AKI [10]. These results are consistent with post-mortem studies showing parasite sequestration and mononuclear cell infiltration in glomerular and peritubular capillaries [44]. Neutrophil gelatinase-associated lipocalin (NGAL), an early marker of renal tubular damage, was elevated in adults with malaria-associated AKI [10]. Oxidative stress and injury due to cell-free haemoglobin and haem from malaria-induced haemolysis may also contribute to tubular damage in malaria. In children with severe malaria, an increase in the haem to haemopexin ratio was observed over hospitalization in children with severe AKI (Stage 3 AKI) [26]. In adults with severe malaria, reduced red blood cell deformability, and increased cell-free haemoglobin and lipid peroxidation (indicative of oxidative stress) were associated with AKI [45].

While the mechanisms leading to severe malaria-associated AKI are not well understood, it is likely a heterogeneous syndrome characterized by reduced renal blood flow due to dehydration, hypotension, and impaired microcirculatory function as a result of parasite sequestration and reduced bioavailable nitric oxide. A subset of children may be at risk of developing direct tubular damage as a result of prolonged ischaemia, endothelial activation, inflammation, and cell-free haemoglobin and haem-mediated injury. In this cohort, an increase in CHI3L1 was associated with significant increases in creatinine, Cystatin C, and BUN by linear regression, and the relationship between CHI3L1 and Cystatin C remained significant following adjustment for creatinine. CHI3L1 levels also correlated with markers of immune activation (CRP, sTREM-1, CXCL10/IP-10), endothelial activation (Ang-2, sICAM-1), and haemolysis (LDH, haem, haemopexin), pathways of injury that are well described in paediatric severe malaria [26–28, 46–48]. As CHI3L1 is produced by tubular cells in response to injury and remodelling [35], it may represent a novel biomarker of AKI in pediatric severe malaria. Additional studies are needed to delineate between CHI3L1 as a marker of inflammation versus AKI by comparing CHI3L1 levels to other established biomarkers of kidney injury (e.g. NGAL) that are well characterized in association with changes to kidney function. Further, additional research is needed to evaluate CHI3L1 over time as it relates to renal recovery and repair in both plasma and urine.

In this study elevated CHI3L1 levels at admission were a risk factor for in-hospital and all-cause 6 month mortality independent of kidney function and treatment group. These results are consistent with reports from adults where CHI3L1 is an independent predictor of all-cause mortality in type II diabetes [49, 50], heart failure [51], and sepsis [52]. Although CHI3L1 was strongly associated with AKI, which is an established risk factor for mortality in severe malaria, the relationship between CHI3L1 and increased risk of death was independent of AKI status suggesting CHI3L1 is not simply a biomarker of kidney function.

This study has several strengths including a randomized trial design with detailed clinical follow up and daily assessment of renal function and plasma CHI3L1 levels. Further, the majority of children were followed up to 6 months allowing us to evaluate the association between CHI3L1 and post-discharge mortality in children with severe malaria. Limitations include a lack of data on renal recovery and long-term renal function in the children. Further, urine was not collected to evaluate plasma versus urine levels of CHI3L1.

## Conclusions

In this study, CHI3L1 was validated as an independent biomarker of morbidity and mortality in children with severe malaria that is associated with the presence and severity of AKI. This provides further evidence that AKI is an important complication in children with severe malaria associated with endothelial activation and inflammation. Additional studies to evaluate the long-term implications of AKI on kidney function in surviving children are urgently needed.

## Additional file

**Additional file 1.** Dataset for malaria, CHI3L1, acute kidney injury, and mortality.

## Abbreviations

AKI: acute kidney injury
Ang-2: angiopoietin-2
GFR: glomerular filtration rate
KDIGO: kidney disease: improving global outcomes
NO: nitric oxide
iNO: inhaled nitric oxide
CHI3L1: chitinase-3-like 1
ARDS: acute respiratory distress syndrome
RDT: rapid diagnostic test
HRP2: histidine rich protein 2
pLDH: pan lactate dehydrogenase
ELISA: enzyme linked immunosorbent assay
LDH: lactate dehydrogenase
EDTA: ethylenediaminetetraacetic acid
LME: linear mixed effects
LTFU: lost to follow up

## References

[1] Sypniewska P, Duda JF, Locatelli I, Althaus CR, Althaus F, Genton B (2017). Clinical and laboratory predictors of death in African children with features of severe malaria: a systematic review and meta-analysis. BMC Med.

[2] von Seidlein L, Olaosebikan R, Hendriksen IC, Lee SJ, Adedoyin OT, Agbenyega T (2012). Predicting the clinical outcome of severe falciparum malaria in african children: findings from a large randomized trial. Clin Infect Dis.

[3] Jallow M, Casals-Pascual C, Ackerman H, Walther B, Walther M, Pinder M (2012). Clinical features of severe malaria associated with death: a 13-year observational study in the Gambia. PLoS ONE.

[4] Kapoor K, Gupta S (2012). Malarial acute kidney injury in a paediatric intensive care unit. Trop Doct.

[5] Imani PD, Odiit A, Hingorani SR, Weiss NS, Eddy AA (2013). Acute kidney injury and its association with in-hospital mortality among children with acute infections. Pediatr Nephrol.

[6] Conroy AL, Hawkes M, Elphinstone RE, Morgan C, Hermann L, Barker KR (2016). Acute kidney injury is common in pediatric severe malaria and is associated with increased mortality. Open Forum Infect Dis.

[7] Trang TT, Phu NH, Vinh H, Hien TT, Cuong BM, Chau TT (1992). Acute renal failure in patients with severe falciparum malaria. Clin Infect Dis.

[8] Dondorp A, Nosten F, Stepniewska K, Day N, White N (2005). Artesunate versus quinine for treatment of severe falciparum malaria: a randomised trial. Lancet.

[9] Mishra SK, Dietz K, Mohanty S, Pati SS (2007). Influence of acute renal failure in patients with cerebral malaria—a hospital-based study from India. Trop Doct.

[10] Plewes K, Royakkers AA, Hanson J, Hasan MM, Alam S, Ghose A (2014). Correlation of biomarkers for parasite burden and immune activation with acute kidney injury in severe falciparum malaria. Malar J.

[11] WHO (2016). World malaria report 2016.

[12] Yeo TW, Lampah DA, Gitawati R, Tjitra E, Kenangalem E, Granger DL (2008). Safety profile of l-arginine infusion in moderately severe falciparum malaria. PLoS ONE.

[13] Hawkes MT, Conroy AL, Opoka RO, Hermann L, Thorpe KE, McDonald C (2015). Inhaled nitric oxide as adjunctive therapy for severe malaria: a randomized controlled trial. Malar J.

[14] Mwanga-Amumpaire J, Carroll RW, Baudin E, Kemigisha E, Nampijja D, Mworozi K (2015). Inhaled nitric oxide as an adjunctive treatment for cerebral malaria in children: a phase ii randomized open-label clinical trial. Open Forum Infect Dis.

[15] Bangirana P, Conroy AL, Opoka RO, Hawkes MT, Hermann L, Miller C, Namasopo S, Liles WC, John CC, Kain KC (2018). Inhaled nitric oxide and cognition in pediatric severe malaria: a randomized double-blind placebo controlled trial. PLoS ONE.

[16] McMahon TJ, Doctor A (2006). Extrapulmonary effects of inhaled nitric oxide: role of reversible S-nitrosylation of erythrocytic hemoglobin. Proc Am Thorac Soc.

[17] Adhikari NK, Burns KE, Friedrich JO, Granton JT, Cook DJ, Meade MO (2007). Effect of nitric oxide on oxygenation and mortality in acute lung injury: systematic review and meta-analysis. BMJ.

[18] Ruan SY, Huang TM, Wu HY, Wu HD, Yu CJ, Lai MS (2015). Inhaled nitric oxide therapy and risk of renal dysfunction: a systematic review and meta-analysis of randomized trials. Crit Care.

[19] Ohno M, Bauer PO, Kida Y, Sakaguchi M, Sugahara Y, Oyama F (2015). Quantitative real-time PCR analysis of YKL-40 and its comparison with mammalian chitinase mRNAs in normal human tissues using a single standard DNA. Int J Mol Sci.

[20] Schmidt IM, Hall IE, Kale S, Lee S, He CH, Lee Y (2013). Chitinase-like protein Brp-39/YKL-40 modulates the renal response to ischemic injury and predicts delayed allograft function. J Am Soc Nephrol.

[21] Erdman LK, Petes C, Lu Z, Dhabangi A, Musoke C, Cserti-Gazdewich CM (2014). Chitinase 3-like 1 is induced by *Plasmodium falciparum* malaria and predicts outcome of cerebral malaria and severe malarial anaemia in a case-control study of African children. Malar J.

[22] Conroy AL, Hawkes M, Hayford K, Hermann L, McDonald CR, Sharma S (2016). Methemoglobin and nitric oxide therapy in Ugandan children hospitalized for febrile illness: results from a prospective cohort study and randomized double-blind placebo-controlled trial. BMC Pediatr.

[23] Shephard MD (2011). Point-of-care testing and creatinine measurement. Clin Biochem Rev.

[24] Schwartz GJ, Munoz A, Schneider MF, Mak RH, Kaskel F, Warady BA (2009). New equations to estimate GFR in children with CKD. J Am Soc Nephrol.

[25] Kidney Disease: Improving Global Outcomes (KDIGO), Acute Kidney Injury Work Group (2012). KDIGO clinical practice guideline for acute kidney injury. Kidney Int.

[26] Elphinstone RE, Conroy AL, Hawkes M, Hermann L, Namasopo S, Warren HS (2016). Alterations in systemic extracellular heme and hemopexin are associated with adverse clinical outcomes in ugandan children with severe malaria. J Infect Dis.

[27] Conroy AL, Hawkes M, McDonald CR, Kim H, Higgins SJ, Barker KR (2016). Host biomarkers are associated with response to therapy and long-term mortality in pediatric severe malaria. Open Forum Infect Dis.

[28] Erdman LK, Dhabangi A, Musoke C, Conroy AL, Hawkes M, Higgins S (2011). Combinations of host biomarkers predict mortality among Ugandan children with severe malaria: a retrospective case-control study. PLoS ONE.

[29] R Core Team (2017). R: a language and environment for statistical computing.

[30] Bates D (2015). Fitting linear mixed-effects models using lme4. J Stat Softw.

[31] World Health Organization (2014). Severe malaria. Trop Med Int Health..

[32] De Loor J, Decruyenaere J, Demeyere K, Nuytinck L, Hoste EAJ, Meyer E (2016). Urinary chitinase 3-like protein 1 for early diagnosis of acute kidney injury: a prospective cohort study in adult critically ill patients. Crit Care.

[33] Hall IE, Stern EP, Cantley LG, Elias JA, Parikh CR (2014). Urine YKL-40 is associated with progressive acute kidney injury or death in hospitalized patients. BMC Nephrol.

[34] Maddens B, Ghesquiere B, Vanholder R, Demon D, Vanmassenhove J, Gevaert K (2012). Chitinase-like proteins are candidate biomarkers for sepsis-induced acute kidney injury. Mol Cell Proteom.

[35] Puthumana J, Hall IE, Reese PP, Schroppel B, Weng FL, Thiessen-Philbrook H (2017). YKL-40 associates with renal recovery in deceased donor kidney transplantation. J Am Soc Nephrol.

[36] Lee CG, Da Silva CA, Dela Cruz CS, Ahangari F, Ma B, Kang MJ (2011). Role of chitin and chitinase/chitinase-like proteins in inflammation, tissue remodeling, and injury. Annu Rev Physiol.

[37] Lee CG, Hartl D, Lee GR, Koller B, Matsuura H, Da Silva CA (2009). Role of breast regression protein 39 (BRP-39)/chitinase 3-like-1 in Th2 and IL-13-induced tissue responses and apoptosis. J Exp Med.

[38] Sohn MH, Kang MJ, Matsuura H, Bhandari V, Chen NY, Lee CG (2010). The chitinase-like proteins breast regression protein-39 and YKL-40 regulate hyperoxia-induced acute lung injury. Am J Respir Crit Care Med.

[39] Weinberg JB, Volkheimer AD, Rubach MP, Florence SM, Mukemba JP, Kalingonji AR (2016). Monocyte polarization in children with falciparum malaria: relationship to nitric oxide insufficiency and disease severity. Sci Rep.

[40] Pozzuoli A, Valvason C, Bernardi D, Plebani M, Fabris Monterumici D, Candiotto S (2007). YKL-40 in human lumbar herniated disc and its relationships with nitric oxide and cyclooxygenase-2. Clin Exp Rheumatol.

[41] Konradsen JR, James A, Nordlund B, Reinius LE, Soderhall C, Melen E (2013). The chitinase-like protein YKL-40: a possible biomarker of inflammation and airway remodeling in severe pediatric asthma. J Allergy Clin Immunol.

[42] Abd El Dayem Soha M, Battah Ahmed A, El Shehaby A, Abd Allah N (2015). Assessment of human cartilage glycoprotein 39 (YKL-40), preptin, and nitric oxide in adolescent patients with type 1 diabetes and its relation to cardiorenal affection. J Pediatr Endocrinol Metab.

[43] Jafari B, Elias JA, Mohsenin V (2014). Increased plasma YKL-40/chitinase-3-like-protein-1 is associated with endothelial dysfunction in obstructive sleep apnea. PLoS ONE.

[44] Nguansangiam S, Day NP, Hien TT, Mai NT, Chaisri U, Riganti M (2007). A quantitative ultrastructural study of renal pathology in fatal *Plasmodium falciparum* malaria. Trop Med Int Health.

[45] Plewes K, Kingston HWF, Ghose A, Maude RJ, Herdman MT, Leopold SJ (2017). Cell-free hemoglobin mediated oxidative stress is associated with acute kidney injury and renal replacement therapy in severe falciparum malaria: an observational study. BMC Infect Dis.

[46] Conroy AL, Glover SJ, Hawkes M, Erdman LK, Seydel KB, Taylor TE (2012). Angiopoietin-2 levels are associated with retinopathy and predict mortality in Malawian children with cerebral malaria: a retrospective case-control study. Crit Care Med.

[47] Lovegrove FE, Tangpukdee N, Opoka RO, Lafferty EI, Rajwans N, Hawkes M (2009). Serum angiopoietin-1 and -2 levels discriminate cerebral malaria from uncomplicated malaria and predict clinical outcome in African children. PLoS ONE.

[48] Elphinstone RE, Riley F, Lin T, Higgins S, Dhabangi A, Musoke C (2015). Dysregulation of the haem-haemopexin axis is associated with severe malaria in a case-control study of Ugandan children. Malar J.

[49] Persson F, Rathcke CN, Gall MA, Parving HH, Vestergaard H, Rossing P (2012). High YKL-40 levels predict mortality in patients with type 2 diabetes. Diabetes Res Clin Pract.

[50] Lin CH, Li HY, Jiang YD, Chang TJ, Chuang LM (2013). Plasma YKL-40 predicts 10-year cardiovascular and all-cause mortality in individuals with type 2 diabetes. Clin Endocrinol (Oxf).

[51] Harutyunyan M, Christiansen M, Johansen JS, Kober L, Torp-Petersen C, Kastrup J (2012). The inflammatory biomarker YKL-40 as a new prognostic marker for all-cause mortality in patients with heart failure. Immunobiology.

[52] Kornblit B, Hellemann D, Munthe-Fog L, Bonde J, Strom JJ, Madsen HO (2013). Plasma YKL-40 and CHI3L1 in systemic inflammation and sepsis-experience from two prospective cohorts. Immunobiology.

